# Medicalization of Sport? A Mixed-Method Study on the Use of Medications in Elite Ice Hockey

**DOI:** 10.3390/sports12010019

**Published:** 2024-01-05

**Authors:** Sofie Christensen, Astrid Gjelstad, Ingunn Björnsdottir, Fredrik Lauritzen

**Affiliations:** 1 Science and Medicine, Anti-Doping Norway, 0855 Oslo, Norwayastrid.gjelstad@antidoping.no (A.G.); 2 Department of Pharmaceutics and Social Pharmacy, School of Pharmacy, University of Oslo, 0316 Oslo, Norway; ingunn.bjornsdottir@farmasi.uio.no; 3 Department of Pharmaceutical Chemistry, School of Pharmacy, University of Oslo, 0316 Oslo, Norway

**Keywords:** ice hockey, athlete, sport, medicalization, antidoping, analgesics, hypnotics, pharmaceuticals

## Abstract

Ice hockey is a high-risk sport known for its dominant macho culture. The purpose of this study was to examine experiences surrounding medication use among male, elite ice hockey players in Norway. A mixed-method design was employed, which first examined medications registered on doping control forms (DCFs) (*n* = 177) and then involved semi-structured focus group interviews (*n* = 5) with elite athletes (*n* = 25). Overall, 68% of the DCFs contained information about ≥1 medication. Among the most registered medications were NSAIDs and hypnotics (20% and 19% of all DCFs, respectively). During the interviews, numerous athletes reported using analgesics to manage injuries and pain caused by the sport, often being motivated by sacrificing themselves for the team during important matches and playoffs. Hypnotics were used due to high cumulative stress due to heavy training and competition load, late-night matches, and playing in a semi-professional league. Athlete support personnel (ASP), including physicians and trainers, were the athletes’ main sources of information. The athletes often displayed a profound and non-critical trust in the advice and products provided to them by their team physician. The findings indicate that male, elite ice hockey players, through their excessive and somewhat ignorant use of medications, expose themselves to health risks and inadvertent doping.

## 1. Introduction

Health problems caused by sport participation and the consecutive use of medications to treat injuries and illnesses have received increased attention in recent years. For example, it has been reported that Finnish athletes use non-steroidal anti-inflammatory drugs (NSAIDs), antibiotics, anti-asthmatic medications, and anti-allergic medications significantly more frequently than a representative sample of age-matched controls [1]. Furthermore, 69% of professional football players competing in the World Cups between 2002 and 2014 used medications during the competition, while more than half used NSAIDs, despite the potential side effects of these drugs, including prolonged recovery after matches [2]. In a recent study on Danish elite athletes, 93% had experience with using sport-related pain-relieving products [3]. Another study found that young male ice hockey players were more prone to using analgesics compared to the control group [4]. Recently, a mapping of Norwegian athletes who had participated in doping controls conducted by Anti-Doping Norway (ADNO) in the period of 2015–2019 showed that 56% of all athletes declared current use of at least one medication on the doping control form [5]. Other studies have reported athletes using analgesics before a competition to avoid pain from possible injuries [6] and hypnotics to cope with symptoms of jet lag [7]. 

In some cases, an increased use of medications among athletes may be justified. Athletes are, in general, more susceptible to various conditions, such as injuries [8], sleeping problems [9], and asthma [10], compared to the rest of the population. In other contexts, medication use may be more related to sport participation and performance than to health aspects. For example, the higher prevalence of oral antibiotics among athletes compared to non-athletes (2.7% vs. 1.3%) [1] could possibly be ascribed to physicians prescribing antibiotics to athletes to expedite their return to sport. Claims that human conditions caused by sport participation are considered and treated as medical problems appear regularly [11]. This concept of medicalization of sport has been linked to the fairly recent development of sport medicine as a specialized field, where athletes utilize medical treatments to enhance athletic performance [12]. 

The World Anti-Doping Agency (WADA) annually publishes a list of substances and methods that are prohibited in sports [13]. Many of the substances on the Prohibited List are found to be active pharmaceutical ingredients in legal medications. Careless use of medications among athletes subjected to a doping control regime may thus lead to positive doping tests and, consequently, an anti-doping rule violation (ADRV) if the athlete has not obtained a therapeutic use exemption (TUE) in line with the regulations of the World Anti-Doping Program. Extensive use of medications may also be indirectly linked to anti-doping, as it has been suggested that it may be related to doping attitudes [14]. Medications are therefore among the many components that make up the athletes’ exposome, thus potentially causing adverse analytical findings [15]. Being vigilant when using medications should therefore be part of active athletesߣ everyday lives. 

The amount and type of medications used vary significantly between sports (see, e.g., [5]). Perhaps due to the high-risk potential and culture [16] and a “tough guy” mentality, ice hockey players may be among the athletes most exposed to using medications to cope with their sport [17]. Recently, several articles have shed light on the problematic relationship with medications among professional ice hockey players, most notably in the National Hockey League [18,19,20]. Moreover, in a recent study of Norwegian athletes, national-level (NL) ice hockey players more often reported using medications than NL athletes in general and compared to athletes in other ball and team sports [5]. Ice hockey players also reported the highest use of hypnotics among all sports.

Greater knowledge about the motivations and rationale for why athletes are using medications, their understanding of risks, and interactions with fellow athletes and with athlete support personnel in their decision-making process is important to protect the health of the athletes, both by contributing to rational use of medications in the sport and to develop tailored anti-doping education aiming to reduce the risk of inadvertent doping. By combining quantitative data from doping control forms with focus group interviews, this mixed-method study seeks to obtain in-depth insight into the prevalence of medication use among male, elite ice hockey players, as well as related attitudes and behaviors. 

## 2. Methods

### 2.1. Quantitative Data from Doping Control Forms

According to the WADA International Standard for Testing and Investigations [21], all athletes who are selected for doping testing must register on the DCF all medications and supplements taken within the previous seven days before sample collection. These data can be organized and used to obtain an overview of the prevalence and trends in the use of these products among athletes, as has been shown in previous studies by our group [5,22]. 

Quantitative data on the use of medications among elite ice hockey athletes were extracted from a database previously developed by ADNO containing information provided by athletes on doping control forms (DCFs) in connection with doping controls performed by ADNO as part of the national testing program in Norwegian sport in the period of 2015–2019. For each DCF, the following information was registered: sport, sex, age group, athlete level, and, if applicable, any medication and/or dietary supplement used by the athlete. No personal information from the DCF was included in the database. Medications were classified according to the Anatomical Therapeutic Chemical (ATC) Classification System. A detailed description of how the database was developed is provided elsewhere [5].

For the present study, data from male, national-level ice hockey athletes were extracted from the database and analyzed. National-level ice hockey players are defined by ADNO as athletes either participating in the senior national team and/or in the top national ice hockey division. 

Continuous data and categorical variables are presented as mean ± SD and percentages, respectively.

### 2.2. Qualitative Data: Experimental Approach and Participants

Quantitative data gathered from the DCFs were used as the basis for developing a semi-structured interview guide (Supplementary Table S1). Qualitative interviews in the form of focus groups were conducted to gain in-depth insight into and contextual understanding of the culture of medication among Norwegian, male, elite ice hockey players. In addition to questions related to medications, the athletes were challenged to also reflect on dietary supplement use and their perception of the national anti-doping agency; however, these results will not be presented here. 

The recruitment of ice hockey teams and athletes for the interviews was conducted in collaboration and in agreement with the Norwegian Ice Hockey Federation. Athletes included in this study had to be male ice hockey players aged 18–50 and currently active in one of the ice hockey teams in the top national ice hockey division. Five teams from the top division were selected based on geographical spread and availability. Contact was set up with the sports director or medical care representatives from selected clubs, who further invited athletes to participate in the focus groups. A group size of four to six athletes was selected for each interview to stimulate discussions and interactions between all participants, where each participant was involved in producing data [23]. 

The interview guide included questions and topics about practices and attitudes related to nutritional supplements, medications, and the general anti-doping effort. Two pilot interviews were conducted before the first actual focus group interview. These pilot interviews involved recreational football athletes and CrossFit athletes, respectively. Following the pilot interviews, minor revisions and clarifications were made to the interview guide. For consistency, the same interviewer, supported by an assistant, conducted all of the interviews. 

A total of 25 male, active ice hockey players aged 20–36 representing five different teams competing in the Norwegian elite ice hockey league (i.e., “Fjordkraftligaen”) participated in this study. The following athletes (A) took part in each focus group: group 1, A1–A6; group 2, A7–A12; group 3, A13–A16; group 4, A17–A21; and group 5, A22–A25.

The interviews were conducted in-season during November and December 2022 at the home arena of each team. The interviewer sought to create an atmosphere where the interview was perceived similarly to normal “café talk”. Each focus group interview lasted for 50–65 min. Data collection was considered complete when no further information was yielded in response to the questions. Data saturation was reached after five focus group interviews, which is in line with previous research on relatively homogenous study populations [24].

### 2.3. Qualitative Data Analysis

The interviews were digitally recorded and transcribed ad verbatim. With the use of Braun and Clarkes’ six-phase method, the entire transcript of 127 pages was thematically analyzed to identify patterns across the data [25]. An inductive and descriptive approach was chosen to analyze the data, where citations from the transcript formed codes, subthemes, and main themes (Table 1). To ensure trustworthiness and increase the validity of this study, the analytic process was discussed with another researcher. Because the language used during the interviews was Norwegian, all direct quotes have been translated into English by the authors. Minor adjustments in wording were made for some of the quotes to increase understanding without changing the meaning of the statements. 

### 2.4. Ethical Considerations

The quantitative part of this study was approved by the Norwegian Regional Committees for Medical Research Ethics (ID 29318), and it was also approved by and registered in the Norwegian Center for Research Data (reference number 241968). The qualitative part of this study was registered in and approved by the Norwegian Center for Research Data (reference number 642391). Before taking part in the focus group interviews, each participant signed a declaration of voluntary informed consent and was given the opportunity to withdraw their contribution until the analysis was completed. In particular, the participants were informed that their data would be anonymized and treated confidentially.

## 3. Results

### 3.1. Quantitative Results—Doping Control Forms

**Figure 1:**
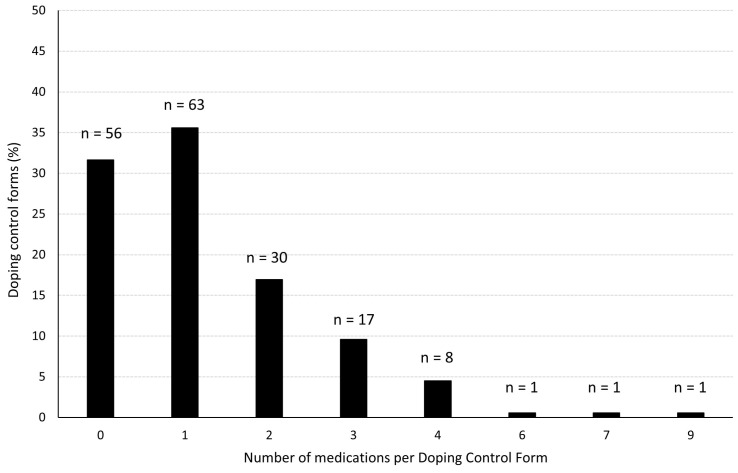
Distribution of the number of medications per doping control form.

For the period of 2015–2019, a total of 177 DCFs were registered from male, national-level athletes as part of ADNO’s national testing program. Of these, 121 (68%) DCFs included information about at least one medication. The maximum number of medications on a single DCF was nine (Figure 1). When including all DCFs (i.e., including DCFs without any registered medication), the mean number of medications per form was 1.29 ± 1.38. When only including DCFs with ≥1 medications, the mean number of medication per form was 1.88 ± 1.29. 

The types of medications appearing most often on the DCFs were ATC code N02B—Other analgesics and antipyretics (*n* = 39 of 177, 22% of all DCFs), ATC code M01A—*NSAIDs* (*n* = 36, 20%), and ATC code N05C—Hypnotics and sedatives (*n* = 34, 19%) (Table 2). The ATC code N05C concerns N05CF – Benzodiazepine related drugs, exclusively. 

### 3.2. Qualitative Results—Focus Group Interviews

#### 3.2.1. Use of Analgesics

The main theme use of analgesics produced four associated subthemes: macho culture; removing pain before, during, and after matches; seasonal-dependent use; and access to analgesics (Table 3).

Certain statements, such as “you always take painkillers before you stop playing” (A11), and certain attitudes, such as “itߣs not dangerous to feel a little pain” (A3), were common. As one athlete (A11) admitted, he probably should not compete with all of the injuries he has had; however, he always ends up playing anyway. He later explained the ice hockey culture in this way:

*“Yes, itߣs a bit macho and loose […] But I think the social environment in hockey is a bit like you keep going until it simply doesnߣt work anymore. You donߣt want to lose your position on the team.”*

(Athlete 11)

One athlete (A7) shared a story from his youth when he once played with a broken arm, even though it hurt; he played and proved how tough he was. Another athlete (A10) described how young ice hockey players could look at the older players on the team and recognize that it is tough to play with injuries and to make sacrifices; it is an integral part of the culture:

*“There is a bit of that macho reputation, and the sport also has a bit of that macho reputation. So, I remember even from when I was younger that it was almost tough to play injured, in a way. Then you could almost brag about it to teammates and such: I have an injury, but Iߣm playing.”*

(Athlete 10)

According to several athletes, painkillers were described as being used to manage pain and injuries and to get ready to compete. As one athlete (A5) explained, if you need them (i.e., painkillers) to be able to compete, you can always take them before matches. Another athlete (A11) reported that instead of missing out on matches, he could use painkillers. Similarly, in two other focus groups, athletes explained that one could take painkillers to be able to continue playing while injured just to get through the match (A14), and that painkillers, such as paracetamol and ibuprofen, were widely used in intense periods (A22). Another athlete explained why he used painkillers:

*“To get rid of some pain and inflammation and things like that. Players take a lot of impact damage. And as “athlete 22” says, when there are a lot of matches and such, there is something you can take to get ready. If it’s next Saturday (the match), there might be quick solutions to things.”*

(Athlete 24)

The use of local anesthetics was identified in three of the focus groups. An athlete (A25) shared that several times, he had seen players receiving injections before a match to manage their injuries, and that injections were a way for to get ready to play. Another athlete (A12) commented that athletes sometimes got injected with local anesthetics if they had suffered a fracture in the playoffs. The dialogue between an athlete and the interviewer provides an inside look at this experience:

*“Interviewer: Yes, where have you come across local anesthetics?*

*Athlete 17: No, so itߣs something you can take if youߣve gotten an injury. So simply just to play without too much pain. Then you take it. Itߣs quite common, for example, during playoffs and in similar situations. One tries to maybe avoid it during regular league matches. But in the playoffs, itߣs, I think itߣs almost, that it exists in all teams, that everyone plays a bit half-injured, so maybe you put a syringe in the shoulder or in the legs just to play if you have gotten shot (by the hockey puck) there.*

*Interview: Mm. How is this done? Is it done on the sidelines or after the match?*

*Athlete 17: No, it is done before, so that the effect occurs at the right time, so itߣs done before or right after the warm-up or during the break and so on. Since we play three periods, we always have 18-min breaks where it can be done.*

*Interviewer: Yes, is it done nowadays or in the past?*

*Athlete 17: No, itߣs still being done, yes.”*

One athlete (A6) said that in elite sports, you do not have time to go to rehabilitation if you want to keep your place on the team. An athlete (A24) shared that he experienced an injury during the COVID-19 pandemic; however, due to the absence of competitions during this period, he was able to take the necessary time to rest and recover. In a normal season, this situation would have caused him significant stress. Another athlete (A16) explained that out of season you have the time to conduct alternative training if injured. However, during the season, it was normal to use painkillers to get through pain. One athlete (A21) explained the use of painkillers just to play without too much pain, especially in the playoffs. One athlete said that it could be that they overuse painkillers for longer periods, especially in the playoffs:

*“Toward the playoffs, the threshold for not playing will be extremely high […] If there are six weeks left, you persevere. So, if you have an upcoming surgery after that, if you use somewhat stronger tablets for the six weeks, such a long period before the surgery, then the risk of getting addicted is present. Especially for tramadol and the tablets we are talking about here. But itߣs not very often, but you hear about it sometimes… But there are exceptions. That players are abusing and overusing these drugs during such periods probably happens more often than in other sports because these periods are so intense. Maybe more often in ice hockey than in other sports because itߣs so intense.”*

(Athlete 6)

One athlete (A21) said that diclofenac was previously known as “hockey candy” among the players, and that its use was still prevalent today. In addition to the use of paracetamol and ibuprofen, A11 mentioned using a combination medicine with esomeprazole and naproxen for a three-week period when the pain was so bad. Athlete 12 mentioned using diclofenac for longer periods, particularly during the playoffs, while athlete 11 had experiences with codeine. In a response to A11, A9 pointed out that it (i.e., codeine) was not easily accessible but could be obtained through Dr. (Name). 

#### 3.2.2. Use of Hypnotics

The main theme *use of hypnotics* produced three subthemes: taboo to talk about; performance; and reasons for use (Table 1). 

Regarding sleeping pills, the athletes reported: “I believe many use it” (A7), “I think it is quite widespread, I have heard of a lot” (A24), and “I understand that some take it to fall asleep” (A9). In three of the focus groups, the athletes themselves brought up the topic of sleeping pills. One athlete (A4) interrupted the talk about painkillers and introduced sleeping pills as a problem in ice hockey:

*“Iߣve seen some who has used a lot of sleeping pills. Thatߣs probably what sticks out about ice hockey when I think back. Painkillers are ok, I think. But when you start taking sleeping pills, that is worse.”*

(Athlete 4)

The same athlete (A4) later described how he recalled players asking for sleeping pills in periods with a lot of matches, and that athletes had been using them for other reasons than sleep, getting addicted, and using them together with alcohol. The non-sleep-related use of sleeping pills was supported by another athlete in the same group (A6). Similarly, athletes A17–A21 explained more about abuse, misuse, and overuse of sleeping pills among teammates. One athlete (A18) explained that athletes he had seen using sleeping pills, such as, for example zopiclone, used them as a recreational drug to get high. The problem of misuse was also brought up by A17:

*“We have seen examples of that, yes. Eh, weߣve had teammates who today struggle with heavier substances. So, Iߣve seen that.”*

(Athlete 17)

Several athletes also mentioned possible adverse effects resulting from the use of sleeping pills. An athlete (A6) provided a rationale for the use of sleeping pills, saying that due to the limited time between matches, training, and normal life, some may need sleeping pills to calm down. At the same time, he acknowledged the importance of only using sleeping pills to aid sleep and not for other purposes. The athletes also discussed the issue of availability. One athlete (A3) pointed out that it is important not to keep a large quantity of tablets at home, as this could potentially increase the risk of abuse. Another athlete (A17) explained that if you get packages of 10, 30, or 100 tablets, it is more likely to trigger abuse. The same athlete talked about the availability of physicians prescribing the medications and distinct cultures between clubs:

*“It has often been like that, itߣs been a problem with it in (country), but the team physicians have been strict about it. Then you have played in some clubs, you know some physicians, and of course there are some who have been a bit sloppy with it who can give prescriptions elsewhere. Itߣs been a big problem in hockey in general, at least in the leagues Iߣve played. But I havenߣt even seen it here, to be completely honest. But I know there have been challenges associated with this in the past.”*

(Athlete 17)

During the discussion about hypnotics, A25 shared experiences from several years ago, when they could simply ask for a tablet after a match and receive it. He noted that today you will be met with more questions about use before you get it. At the same time, another athlete (A17) admitted that: “I know that itߣs used a lot more in other teams in Norway, Iߣll be honest about that”. 

One athlete (A8) drew a comparison between coming home after a late-night match and coming home after a party. The athletes named various stressful factors, such as a tight match program, traveling, stress from training, late evening matches, “adrenaline rush”, and the challenges of playing in a semi-professional league, as reasons for sleep problems:

*“Yes, so the fact that itߣs being used is because we often play late matches. Itߣs also a very adrenaline-filled sport. So, itߣs quite a challenge to fall asleep after matches. A lot of people have it. Then itߣs getting up early the next day, and there is often a tight match schedule.”*

(Athlete 21)

Another athlete stated:

*“Instead of getting to the hotel room at 11:30 p.m. and staying up until three oߣclock without getting any sleep, and you have to wake up at 7 or 8 a.m., you take a sleeping pill when you get to the hotel room or something, then youߣll probably fall asleep at midnight or 12:30 a.m. But you donߣt sleep well, because in a way you are ruining the quality of your sleep. But at least you are able to fall asleep.”*

(Athlete 7)

Many athletes emphasized the difficulty of combining late-night games with attending a regular job the morning after. For example:

*“But there are quite a few people who have the problem after matches that they cannot fall asleep, no matter how the match went, because we still have a lot of adrenaline after the match. Letߣs say you play a match at 7 p.m., then youߣre not home until 11 p.m., and you still have some adrenaline, canߣt sleep. Then you have training and work early the next day.”*

(Athlete 14)

One athlete (A6) revealed that it can be difficult for them to calm down after a match, especially when they have limited time to sleep before morning training the next day. To cope with this, he sometimes used sleeping pills. Similarly, another athlete (A3) shared his struggle with sleeping after matches due to the consumption of coffee and energy drinks. That is why he sometimes used sleeping pills. Athlete A16 explained that because of travelling to various places to play games, he used sleeping pills to fall asleep. Another (A7) said that he used sleeping pills because of late-night matches because the body was still “active” when he tried to sleep. 

During the interviews, the athletes were questioned about the higher usage of sleeping pills in ice hockey compared to other sports; in response, the athletes pointed to the tight schedule of matches, which can make it challenging to get enough sleep and restitution. Several athletes (A14, A17, and A25) described ice hockey as more intense than, for example, football, with more matches and less time for restitution. Additionally, several athletes (A14, A21, and A25) suggested that the issue with playing in a semi-professional league could potentially be a contributing factor to this:

*“But then there are perhaps more hockey players than in other team sports who are not quite professional, while in football most are professionals. Here, there are quite a few who have a job on the side. So, yes, but there are a lot of people here who have more on their plate, perhaps. Not getting as much rest.”*

(Athlete 25)

The fact that inadequate sleep could negatively affect performance was raised by multiple athletes (A3, A4, A5, A8, A16, and A25). An athlete (25) expressed the stress and frustration that come with lying in bed throughout the night, unable to fall asleep and knowing that their lack of sleep would negatively affect their performance the following day. 

In one of the focus groups, one athlete explained this as a cultural phenomenon, saying that a team could consist of athletes who did not use sleeping pills, but, over time, everyone starts using sleeping pills. The same athlete described the culture in greater detail:

*“I think itߣs a disadvantage that you have in a team a collection of athletes with such a big difference in age. For example, there may be a culture among some older people who use sleeping pills. So, they do that; take some sleeping pills to be able to sleep because they must go to work the next day or have small children. And then the 17-year-old on the team sees that and thinks “Oh, he takes it, it is cool”, they also start taking it, without even needing it. And then it becomes more likely that they may use it in some form (as a recreational drug) to get high, that is what is dangerous. A large group with a large age difference, so itߣs like that. You can never guarantee that it will be used for what itߣs supposed to be used for when so many get access to it. So, I think itߣs much better that itߣs something that everyone must discuss with their physician, rather than that it is, for example, handed out to everyone.”*

(Athlete 21)

#### 3.2.3. Trust in the Athlete Support Personnel

The main theme trust in the athlete support personnel (ASP) produced three subthemes: the athlete and physician as a “team”, ASP’s competence, and availability of prescribers (Table 2).

One athlete (A21) shared that he relies on his physician to decide whether a medication is necessary for him or not. He trusts that the physician weighs the risks and benefits for using a specific medication on his behalf. Furthermore, he actively does not ask about side effects for the reason that he prefers not to know. Regarding the issue of checking whether a medication is placed on the Prohibited List, more of the athletes share the opinion that their physician is the most knowledgeable and capable person to perform this control. Athlete (17) described that he places full trust in the physician’s evaluation of whether a prescribed medication aligns with the anti-doping regulations. However, he acknowledged that it is his responsibility in the end to ensure the medication is legal due to the Prohibited List. Athlete 21 further added that in his opinion, the physician is more qualified to check the medication than himself, stating that if not, the physician did not do their job. In another focus group, three athletes (A13, A14, and A16) expressed that they were not aware of any other reliable sources of information besides their physician when it comes to deciding whether a medication or product is prohibited or not. Athlete (A18) described a situation:

*“A while ago I was given a new medication, and then our physician checked anti-doping (webpage), and confirmed that it was ok to use, so it could be prescribed. So, then it was up to the physician to check it. So, then we don’t have to also check it once the physician has checked it.”*

(Athlete 18)

When asking the athletes about whether they would have taken a dietary supplement recommended by their physician, the athletes displayed considerable trust in the physician, stating, for example: “I would have asked but taken it” (A23), “We have complete trust in our physician, so we are not worried” (A3, and supported by A1, A2, A4, A5, and A6), “I wouldn’t have discussed it with the physician” (A4 and A5), and “we trust our physicians 100 percent, no matter what” (A24). One athlete discussed the trust in their ASP related to medications by using a recent high-profile doping case in Norwegian sport as an example:

*“They are a team, right. The physician and Johaug (former Norwegian cross-country skier), they are like a team. You trust that person a lot. You (the interviewer) are the one who knows about it, I have no idea, but you (the interviewer) do. Then he (the physician) says that it is ok. Should you go to someone else who is not on the team to verify it? If he (the other physician) knows it’s allowed but also that it can make you better, then he might say no because he doesn’t want you to get better because he’s on a different team, right. Then I would rather completely trust my own physician.”*

(Athlete 14)

Another athlete talked about a time he received medical help from the opposing team’s physician during a match after an acute injury:

*“I actually got some injections. It happened when I played in X (club). He is quite liberal. Haha (laughs). The physician. I was in so much pain, so he gave me three shots, and then I was completely gone. I believe it was something like local anesthesia. I don’t know… It was during a match, so it was the other team’s physician that helped me.”*

(Athlete 24)

Some of the athletes problematized the difference between team sports and individual sports. For example, one athlete said:

*“I can imagine that if it concerns individual sports and there is a new drug they are going to use, they ask the physician right away, whereas here it’s often the case that I had asked the physician and it’s like yes, it’s probably ok. Even if you have easy access to the physician, you could just send a message, but you don’t have the same relationship as individual athletes do.”*

(Athlete 20)

## 4. Discussion

In this study, we combine qualitative and quantitative data to provide insight into the prevalence of use of medications among Norwegian, male, elite ice hockey players and related attitudes and practices. To our knowledge, this is the first study exploring drug use among athletes using a mixed-method approach. 

Overall, the results support the notion that certain parts of sport are becoming medicalized. The quantitative results suggest that medications are relatively commonly used among male, elite ice hockey players, with analgesics, NSAIDs, and hypnotics being the most prevalent medications. In support of this, the focus group interviews revealed the following themes: use of analgesics, use of hypnotics, and trust in the ASP. Athletes reported using analgesics to manage pain from sport-related injuries, often so that the athletes could continue competing despite injuries. Hypnotics were used to aid sleep and recovery in an everyday life characterized by stress caused by traveling, a tight match schedule that often takes place late at night, and other private commitments. The fact that the Norwegian top ice hockey league is a semi-professional league, where many athletes combine being an elite ice hockey player with a part-time, daytime job, amplifies the total load on the players and the challenges many athletes experience in relation to getting enough rest. Some athletes had experienced situations where the use of hypnotics had led to addiction and abuse. Furthermore, many athletes demonstrated a considerable non-critical trust in the medical staff and ASP, which may put them at risk of inadvertent doping.

The use of analgesics among athletes to overcome pain and suppress their body’s signals instead of resting when injured is not a new finding [3,26,27]. Previous studies have reported that 8.1% of Finnish athletes used NSAIDs over a seven-day period [1], and during the Olympic Games in Sydney in the year 2000, it was reported that 25.6% of the DCFs collected from the athletes during the competition included information about NSAIDs [28]. An even higher prevalence has been shown in top-level football tournaments, where 43% of the athletes reported using NSAIDs [2]. In the present study, 20% of the athletes registered NSAIDs on their DCFs, putting elite ice hockey players in the middle of what has been previously reported regarding NSAID use among athletes. 

Previous studies have demonstrated that increased training and competition demands were linked to decreased sleep duration and efficiency [29], and that stress from competition, training, and traveling affected sleep negatively [9]. The athletes in the present study described using hypnotics to minimize the challenges of limited sleep and to ensure sufficient restitution. Compared to the general male population in Norway in the age range of 20–39, the DCFs of ice hockey players in the present study indicate a notably higher use of Z-hypnotics (4.9% vs. 19%) [30]. The fact that some athletes describe misuse and recreational use is not surprising, as the use of Z-hypnotics is associated with a risk of misuse, abuse, dependence, and withdrawal issues [31]. The use of analgesics and hypnotics among athletes illustrates a phenomenon where certain human conditions resulting from intense sport participation can be wrongly attributed to medical issues and treated with medications. 

The well-being and health of the athlete may sometimes conflict with the priorities and interests of the team, as well as the ASP’s role in preventing doping [32]. During the interviews, we found that many athletes discussed situations where they had used over-the-counter medications, such as ibuprofen and paracetamol, or prescription medications, such as tramadol and codeine, after a match to suppress the pain from injuries. Additionally, some athletes received injections of local anesthetics to manage severe injuries and to persevere through crucial matches. From a pharmaceutical perspective, masking pain could potentially worsen the injury, and this is a risk the athletes should be aware of [33]. From a medical perspective, as Orchard describes, there may not be adequate time during a match to discuss the risks associated with using a specific medication with the player or for the player to ask about the risks, which raises concerns regarding autonomy [34]. The athletes are put in a tricky situation where they have to choose between risking their health by playing with injuries and risk losing their spot on the team if they do not sacrifice themselves. This concern extends beyond the use of local anesthetics to include adverse effects of oral anesthetics [6] and addiction to hypnotics [31]. 

Besides obvious health related concerns, excessive use of medications in sport may also have a doping/anti-doping dimension. Most notably, medications may themselves contain active ingredients which are prohibited to use by athletes according to the WADA Prohibited List [13]. Excessive use of medications may thus increase the risk of an athlete’s sample resulting in an adverse analytic finding following a doping test. This risk may be further increased if the athlete lacks knowledge about the potential risk of medications containing prohibited substances, and if they lack routines for checking the content of their medications against the Prohibited List [35]. 

In the present study, there were multiple examples of athletes explaining that they trusted their team’s medical staff, as well as other physicians, to not treat them with or prescribe medications that potentially could contain ingredients on the Prohibited List. However, according to the WADC’s principle of strict liability, it is the athlete’s responsibility to check their medication before using it [36]. In 2016, a former Norwegian Olympic champion, World Cup winner, and world champion tested positive for an anabolic steroid after using an ointment approved for use by the team physician [37]. For the violation, the athlete received an 18-month ban, making her miss the 2018 winter Olympics. The doping case resulted in massive national attention and, consequently, an increased awareness of anti-doping regulations among Norwegian athletes. With this background in mind, it was somewhat surprising that the athletes in this study demonstrated such a profound and non-critical trust in their physician and ASP to control whether a medication was safe to use or not according to the Prohibited List. This finding suggests that the athletes distanced themselves from or were unaware of the principle of strict liability and possible negative consequences for themselves if they are careless when using medications. If the physician had controlled the medication in regard to the anti-doping rules, they believed that they did not need to control it themselves. This is indeed worrisome, as studies have reported a lack of knowledge around doping and the use of medications among physicians [38,39,40].

## 5. Limitations

This study does have some limitations that are worth noting. The results are only representative of the population of elite, male ice hockey players, and they are not necessarily generalizable to other athletes. Additionally, the athletes may have intentionally concealed or withheld certain details, which could have led to a lack of information sharing and affected the accuracy of the results. Also, the quantitative and qualitative datasets were collected during two different time periods (2015–2019 and 2022, respectively), making it possible for them to describe two different cultures. However, the fact that the main findings from the interviews are consistent with what was reported on the DCFs from the same category of athletes some years earlier suggests that the results presented in the present paper reflect the actual culture around the use of medications among male, elite ice hockey players in Norway. 

## 6. Perspective

A key element of any anti-doping education program is to provide information about the safe use of medications and dietary supplements. Still, some athletes demonstrate reckless use of such products, some of which may have a high risk of containing substances on the Prohibited List. In addition, athletes risk harming their health by using medications beyond what is considered to be good practice. The results clearly show that there is a need to strengthen education efforts, particularly in relation to the anti-doping rules (e.g., the principle of strict liability) and the potential risk of using medications. This study also confirms the key role that medical and other athlete support personnel play in the process of medicalizing sport, and that their behaviors may protect but also negatively influence the athlete and put them at increased risk of intentional as well as inadvertent doping.

In this study, athletes often explained that the use of hypnotics in particular was frequently related to factors beyond the athlete’s control, such as late-night games and a busy match schedule. The sport federation should therefore assess the match program as a potential mediating risk factor for inappropriate medication use and consider whether changes should be made to facilitate athletes’ recovery.

This study reveals several interesting issues related to the athletes’ health, including common practices related to the prescription of medications to active athletes, which could be topics of future studies. Physicians and other medical support staff are required to comply with the anti-doping rules, and they play a pivotal role in securing a doping-free sport [41].

## Figures and Tables

**Table 1:** Thematic approach to transcription. The first example demonstrates how one athlete expresses the use of hypnotics and the codes, subthemes, and main themes that are related to this. The next example shows how an athlete talked about relationships with support personnel and macho culture.

Interview Extract	Codes	Subtheme (Main Theme)
Let’s say we play three matches a week, and then we have a match at 7 p.m., so then we don’t finish until 10 p.m., then we don’t get home until 11 p.m., because we have a late dinner. Then, I think there are many, or I understand why people want sleeping pills to be able to sleep.	Tight program Late-night matches Culture	Reasons for use (Use of hypnotics)
I think there may be a little more and closer follow-up in individual sports on a weekly basis about how you feel, so if you have minor injuries, you simply must take that into account. But I think the environment around hockey is a bit like that, that you keep going until it simply doesn’t work.	Individual versus team sports Tolerate pain and injuries	Access to prescribers (Athlete support personnel) Macho culture (Use of analgesics)

**Table 2:** The five most registered medications among male, national-level ice hockey players extracted from doping control forms (DCFs) collected during doping controls performed by Anti-Doping Norway in the period of 2015–2019.

Pharmaceutical Group	ATC * Code	Usage Areas	Count (n) **	% of All DCFs **
Other analgesics and antipyretics	N02B	Pain relief	39	22%
NSAIDs	M01A	Pain relief	36	20%
Hypnotics and sedatives	N05C	Induce, extend, or improve sleep	34	19%
Antihistamines for systemic use	R06A	Allergy	17	9.6%
Decongestants and other nasal preparations for topical use	R01A	Cold, allergy	17	9.6%
Total doping control forms (DCFs) with information about ≥1 medication.			121	68%

* ATC = Anatomical Therapeutic Chemical Classification System. ** Number and percent of DCFs with information about at least one drug from the specific pharmaceutical group. One DCF may contain information about more than one Pharmaceutical group.

**Table 3:** Main themes, subthemes, and codes related to focus group interviews.

Main Theme	Subtheme	Codes
Use of analgesics	Macho culture	Tolerate pain and injury Keep the place on the team It is tough to play injured
	Removing pain before, during, and after matches	Mechanic impact injuries Injury management
	Seasonal-dependent use	Sacrifice your own health More use of analgesics in important parts of the season
	Access to analgesics	The accessibility of a physician Used in important matches
Use of hypnotics	Taboo to talk about	More in other clubs More before Aware of the consequences
	Reasons for use	Traveling Heavy training volume Tight program Late-night matches Adrenaline rush Semi-professional league Culture
	Performance	The importance of sleep as an athlete Afraid of too little sleep
Trust in athlete support personnel (ASP)	Athlete and physician as a team	Common goal Trust between physician and athlete
	ASP’s competence	Control of substances and products Physicians’ competence Division of responsibility
	Availability of prescribers	Several prescribers Individual versus team sports Semi-professional league

## Data Availability

The data presented in this study are available on request from the corresponding author. The data are not publicly available due to privacy and ethical restrictions.

## Supplementary Materials

The following supporting information can be downloaded at: https://www.mdpi.com/article/10.3390/sports12010019/s1, Table S1: Interview guide.

## References

[1] Alaranta A., Alaranta H., Heliövaara M., Airaksinen M., Helenius I. (2006). Ample use of physician-prescribed medications in Finnish elite athletes. Int. J. Sports Med..

[2] Tscholl P.M., Vaso M., Weber A., Dvorak J. (2015). High prevalence of medication use in professional football tournaments including the World Cups between 2002 and 2014: A narrative review with a focus on NSAIDs. Br. J. Sports Med..

[3] Overbye M. (2021). Walking the line? An investigation into elite athletes’ sport-related use of painkillers and their willingness to use analgesics to train or compete when injured. Int. Rev. Sociol. Sport.

[4] Selanne H., Ryba T.V., Siekkinen K., Kyröläinen H., Kautiainen H., Hakonen H., Mikkelsson M., Kujala U.M. (2014). The prevalence of musculoskeletal pain and use of painkillers among adolescent male ice hockey players in Finland. Health Psychol. Behav. Med..

[5] Gjelstad A., Herlofsen T.M., Bjerke A.-L., Lauritzen F., Björnsdottir I. (2023). Use of pharmaceuticals amongst athletes tested by Anti-Doping Norway in a five-year period. Front. Sports Act. Living.

[6] Warden S. (2009). Prophylactic misuse and recommended use of non-steroidal anti-inflammatory drugs by athletes. Br. J. Sports Med..

[7] Manfredini R., Manfredini F., Conconi F. (2000). Standard melatonin intake and circadian rhythms of elite athletes after a transmeridian flight. J. Int. Med. Res..

[8] Drawer S., Fuller C.W. (2002). Evaluating the level of injury in English professional football using a risk based assessment process. Br. J. Sports Med..

[9] Cameron A.F.M., Perera N., Fulcher M. (2021). Professional Athletes Have Poorer Sleep Quality and Sleep Hygiene Compared With an Age-Matched Cohort. Clin. J. Sport Med..

[10] Burns J., Mason C., Mueller N., Ohlander J., Zock J.P., Drobnic F., Wolfarth B., Heinrich J., Omenaas E., Stensrud T. (2015). Asthma prevalence in Olympic summer athletes and the general population: An analysis of three European countries. Respir. Med..

[11] Waddington I. (1996). The Development of Sports Medicine. Sociol. Sport J..

[12] Kundu P. (2018). A Medical Sociological Perspective of Doping in Sports. Int. J. Phys. Educ. Sports Sci..

[13] WADA The Prohibited List. https://www.wada-ama.org/en/prohibited-list.

[14] Melzer M., Elbe A.-M., Strahler K. (2022). Athletes’ use of analgesics is related to doping attitudes, competitive anxiety, and situational opportunity. Front. Sports Act. Living.

[15] Thevis M., Kuuranne T., Fedoruk M., Geyer H. (2021). Sports drug testing and the athletes’ exposome. Drug Test. Anal..

[16] Atkinson M. (2019). Sport and Risk Culture. The Suffering Body in Sport.

[17] Gaetz M. (2022). Substance availability and use in ex-professional ice hockey enforcers. Sci. Rep..

[18] Davies C.R. (2021). Addiction & Substance Abuse in the NHL—It’s Bigger Than the Game. The Hockey Writers.

[19] Nimens R. (2020). Pushing through the pain, NHLers say theyߣre regulalry taking painkillers in order to play. CTV News.

[20] Basu A. (2022). Paul Byron is looking forward to a life without chronic pain, something that is all too rare in hockey. The Athletic.

[21] WADA (2021). International Standard for Testing and Investigations.

[22] Lauritzen F., Gjelstad A. (2023). Trends in dietary supplement use among athletes selected for doping controls. Front. Nutr..

[23] Gill P., Stewart K., Treasure E., Chadwick B. (2008). Methods of data collection in qualitative research: Interviews and focus groups. Br. Dent. J..

[24] Hennink M., Kaiser B.N. (2022). Sample sizes for saturation in qualitative research: A systematic review of empirical tests. Soc. Sci. Med..

[25] Braun V., Clarke V. (2006). Using thematic analysis in psychology. Qual. Res. Psychol..

[26] Roderick M., Waddington I., Parker G. (2000). PLAYING HURT: Managing Injuries in English Professional Football. Int. Rev. Sociol. Sport.

[27] Mayer J., Giel K.E., Malcolm D., Schneider S., Diehl K., Zipfel S., Thiel A. (2018). Compete or rest? Willingness to compete hurt among adolescent elite athletes. Psychol. Sport Exerc..

[28] Corrigan B., Kazlauskas R. (2003). Medication Use in Athletes Selected for Doping Control at the Sydney Olympics (2000). Clin. J. Sport Med..

[29] Conlan G., McLean B., Kemp J., Duffield R. (2022). Effect of Training/Competition Load and Scheduling on Sleep Characteristics in Professional Rugby League Athletes. J. Strength Cond. Res..

[30] The Norwegian Prescription Database. https://www.norpd.no/.

[31] Schifano F., Chiappini S., Corkery J.M., Guirguis A. (2019). An Insight into Z-Drug Abuse and Dependence: An Examination of Reports to the European Medicines Agency Database of Suspected Adverse Drug Reactions. Int. J. Neuropsychopharmacol..

[32] Patterson L.B., Backhouse S.H., Jones B. (2023). The role of athlete support personnel in preventing doping: A qualitative study of a rugby union academy. Qual. Res. Sport Exerc. Health.

[33] Orchard J.W. (2004). Is it Safe to Use Local Anaesthetic Painkilling Injections in Professional Football?. Sports Med..

[34] Orchard J. (2001). The use of local anaesthetic injections in professional football. Br. J. Sports Med..

[35] Mottram D., Chester N., Atkinson G., Goode D. (2008). Athletes’ Knowledge and Views on OTC Medication. Int. J. Sports Med..

[36] WADA (2021). World Anti-Doping Code 2021.

[37] Dunbarap G. (2017). Ski star Johaug banned from 2018 Olympics in doping case. AP News.

[38] Dikic N., McNamee M., Günter H., Markovic S.S., Vajgic B. (2013). Sports physicians, ethics and antidoping governance: Between assistance and negligence. Br. J. Sports Med..

[39] Hughes D., Vlahovich N., Welvaert M., Tee N., Harcourt P., White S., Vernec A., Fitch K., Waddington G. (2020). Glucocorticoid prescribing habits of sports medicine physicians working in high-performance sport: A 30-nation survey. Br. J. Sports Med..

[40] Mazanov J., Backhouse S., Connor J., Hemphill D., Quirk F. (2014). Athlete support personnel and anti-doping: Knowledge, attitudes, and ethical stance. Scand. J. Med. Sci. Sports.

[41] Tandon S., Bowers L.D., Fedoruk M.N. (2015). Treating the elite athlete: Anti-doping information for the health professional. Mo. Med..

