# Supplementary material for: Medicalization of Sport? A Mixed-Method Study on the Use of Medications in Elite Ice Hockey
Source: Sports (Basel). 2024 Jan 5;12(1):19. doi: 10.3390/sports12010019 (PMC10818849; doi:10.3390/sports12010019)
Supplement: Supplementary file 1 [file sports-12-00019-s001.zip › sports-2774885-supplementary.pdf]

Supplementary table S1. – Interview guide

Theme	Questions	Examples of follow-up questions
Introduction	Present the aim of the study.<br>Framework for the focus group interview.<br>General talk about the season, ice hockey, and training.	
Main part	What do you think of when we discuss medications?	
	Can you discuss how you think of the use of medications in sports?	In your sport, are there any medications you think are used more often than in other sports?<br><br>What do you think about the need for the use of medications among athletes and the rest of the population?<br><br>Scenario: The news talks about sports using more analgesics to keep playing or athletes using more hypnotics during busy periods, a) What do you think about this? b) Do you think the situation is different for top and lower-level athletes?
	Where do you find information about medications?	Which assessment do you make for yourself?<br><br>Is there a difference in what you think about what your team physician, your general practitioner, and you can do in terms of assessment?<br><br>Who do you think has the responsibility (in the case of doping)?
	Within the team, do you exchange information about the use of medications?	What do you think of teammates´ use of medications?<br><br>Do the players influence each other in the use of medications?
	If it has not yet been discussed, ask about the use of hypnotics.	
Ending	Does anyone have any more questions or anything they want to say?	
